# RPS27, a sORF-Encoded Polypeptide, Functions Antivirally by Activating the NF-κB Pathway and Interacting With Viral Envelope Proteins in Shrimp

**DOI:** 10.3389/fimmu.2019.02763

**Published:** 2019-12-17

**Authors:** Meng-Qi Diao, Cang Li, Ji-Dong Xu, Xiao-Fan Zhao, Jin-Xing Wang

**Affiliations:** ^1^Shandong Provincial Key Laboratory of Animal Cells and Developmental Biology, School of Life Science, Shandong University, Qingdao, China; ^2^State Key Laboratory of Microbial Technology, Shandong University, Qingdao, China; ^3^Laboratory for Marine Biology and Biotechnology, Qingdao National Laboratory for Marine Science and Technology, Qingdao, China

**Keywords:** short open reading frame (sORF), sORF encoded polypeptides, white spot syndrome virus, antimicrobial peptides, dorsal, Relish, kuruma shrimp

## Abstract

A small open reading frame (smORF) or short open reading frame (sORF) encodes a polypeptide of <100 amino acids in eukaryotes (50 amino acids in prokaryotes). Studies have shown that several sORF-encoded peptides (SEPs) have important physiological functions in different organisms. Many ribosomal proteins belonging to SEPs play important roles in several cellular processes, such as DNA damage repair and apoptosis. Several studies have implicated SEPs in response to infection and innate immunity, but the mechanisms have been unclear for most of them. In this study, we identified a sORF-encoded ribosomal protein S27 (RPS27) in *Marsupenaeus japonicus*. The expression of *MjRPS27* was significantly upregulated in shrimp infected with white spot syndrome virus (WSSV). After knockdown of *MjRPS27* by RNA interference, WSSV replication increased significantly. Conversely, after *Mj*RPS27 overexpression, WSSV replication decreased in shrimp and the survival rate of the shrimp increased significantly. These results suggested that *Mj*RPS27 inhibited viral replication. Further study showed that, after *Mj*RPS27 knockdown, the mRNA expression level of *Mj*Dorsal, *Mj*Relish, and antimicrobial peptides (AMPs) decreased, and the nuclear translocation of *Mj*Dorsal and *Mj*Relish into the nucleus also decreased. These findings indicated that *Mj*RPS27 might activate the NF-κB pathway and regulate the expression of AMPs in shrimp after WSSV challenge, thereby inhibiting viral replication. We also found that *Mj*RPS27 interacted with WSSV's envelope proteins, including VP19, VP24, and VP28, suggesting that *Mj*RPS27 may inhibit WSSV proliferation by preventing virion assembly in shrimp. This study was the first to elucidate the function of the ribosomal protein *Mj*RPS27 in the antiviral immunity of shrimp.

## Introduction

A small open reading frame (smORF) or short open reading frame (sORF) encodes polypeptides of <100 amino acids in eukaryotes (1). SmORFs can encode functional polypeptides, designated as smORF-encoded polypeptides (SEPs or micropeptides), or act as cis-translational regulators (2). Hundreds and thousands of smORF sequences are found in eukaryotic genomes (3). One of the differences between smORF and a traditional ORF is the length of the DNA sequence, i.e., the traditional ORF usually exceeds 100 codons. The second difference is that smORF-encoded SEPs do not need to undergo protease hydrolysis and play direct physiological roles, whereas a traditional peptide, such as an 180 amino acid pre-glucagon, must undergo protease hydrolysis to become the biologically active glucagon (with 29 amino acids) (4, 5).

smORFs play important roles in many fundamental biological processes in human cells, animals, plants, and bacteria (6–8). For example, a new smORF–sarcolamban gene identified in *Drosophila* encodes two novel functional SEPs, namely a 28- and a 29-amino acid peptide homologous to the mammalian 30-amino acid polypeptide of muscle lipoprotein and the 52-amino acid phosphoprotein (9–11). Sarcolamban regulates calcium signaling and muscle contraction. Loss of sarcolamban leads to arhythmia, but its overexpression can lead to increased heart rate in *Drosophila* (9). A smORF-encoded myoregulin (MLN) is a homolog of myosin and phosphoprotein. It can interact with SERCA, the membrane pump that controls muscle relaxation by regulating Ca^2+^ uptake into the sarcoplasmic reticulum. Mice lacking MLN have higher endurance than wild-type mice and can travel farther, suggesting that MLN is an important regulator of skeletal muscle physiology (12). Several reports have implicated SEPs in response to infection and innate immunity. For example, Jackson et al. (13) found that the translation of a new ORF “hidden” within the long noncoding RNA Aw112010 can control mucosal immunity during both bacterial infection and colitis. Virus infection of human lung cancer cells induced 19 novel smORFs in noncoding RNAs either up- or downregulated during infection, suggesting that these smORFs may be immune regulators involved in the antiviral process (14).

Ribosome protein S27 (RPS27) belongs to the 40S subunit of ribosome, also called metallopanstimulin-1 (MPS-1) protein. RPS27 was identified as a growth-factor-inducible gene and encodes an 84-amino acid (9.5 kDa) protein with a zinc finger motif (15–17). Many studies show that some ribosomal proteins have other functions in addition to protein synthesis (18). For example, the ribosomal protein L13a participates in the formation of the complex respiratory syncytial virus-activated inhibitor of translation during respiratory syncytial virus infection, thereby acting as an antiviral agent (19). Ribosomal protein L11 and L23 interact with HDM2 (the human counterpart of murine double minute two gene), and this interaction inhibits the E3 ligase function of HDM2 and stabilizes and activates p53 (20–22). Recent studies have shown that RPS27/MPS-1 is overexpressed in 86% of gastric cancer tissues, and its overexpression is related to tumor nodule metastasis. In gastric cancer cells, the expression of RPS27/MPS-1 affects the NF-κB pathway of gastric cancer cells (23). However, current reports about the function of RPS27 are mostly on human cancers, and reports on other functions are few.

In shrimp, The NF-κB pathways [Toll and immune deficiency (IMD) pathways] play important roles in innate immunity (24, 25). After pathogen infection, pattern recognition receptors (PRRs) recognize the pathogen-associated molecular patterns of invading pathogens and activate NF-κB pathways (Toll and IMD pathways). Consequently, the expression of specific genes regulated by the pathways, such as antimicrobial peptides (AMPs) and C-type lectins, are increased to defend against pathogen invasion (26–29).

White spot syndrome virus (WSSV) is one of the most prevalent, widespread, and lethal viruses and causes great losses in the shrimp aquaculture industry (30). Understanding the molecular mechanism between host–pathogen interactions will contribute significantly to the treatment of this pathogen. In the present study, we identified a sORF-encoded polypeptide, ribosome protein S27, in kuruma shrimp (*Marsupenaeus japonicus*) and denoted it as *Mj*RPS27. We found that *Mj*RPS27 was upregulated in shrimp challenged by white spot syndrome virus (WSSV). RNA interference and overexpression analysis revealed that *Mj*RPS27 had an antiviral function. The possible underlying mechanism was studied.

## Materials and Methods

### Experimental Materials

Kuruma shrimp *Marsupenaeus japonicus* (8–10 g each) were purchased from the fish market in Jinan and Qingdao, Shandong Province, and cultured in a circulating aquaculture system filled with natural seawater before the experiments. The preparation of WSSV inoculum and quantification of viral copy numbers of the inoculum followed our previously described methods (31).

### Viral Challenge and Tissue Collection

Shrimp were randomly divided into two groups (30 shrimp each) for the challenge experiment. One group was intramuscularly injected with 50 μL of WSSV (5 × 10^7^ copies/shrimp) at the penultimate segment of shrimp using a microsyringe, and another group was injected with the same amount of PBS (140 mM NaCl, 2.7 mM KCl, 10 mM Na_2_HPO_4_, and 1.8 mM KH_2_PO_4_; pH 7.4) as a control. Different organs (heart, hepatopancreas, gills, stomach, and intestine) and total hemocytes were collected from shrimp at different time points (0, 6, 12, 24, and 48 h post injection) for RNA extraction. For hemocytes collection, shrimp hemolymph was extracted using a 5 mL syringe preloaded with anticoagulant (0.45 M NaCl, 10 mM KCl, 10 mM EDTA, and 10 mM HEPES; pH 7.45) beforehand at 1:1 hemolymph/anticoagulan ratio. After centrifugation at 800 × *g* for 6 min at 4°C, hemocytes were collected and used to extract RNA or protein for tissue distribution and expression pattern analysis. At least three shrimp were used for a tissue collection.

### RNA Extraction and cDNA Reverse Transcription

The different organs collected from viral-challenged or control shrimp were homogenized on ice with 1 mL of Trizol reagent (Invitrogen, Carlsbad, CA, USA). The collected hemocytes were suspended with Trizol reagent. All homogenates of different organs and hemocytes were centrifuged at 12,000 × *g* for 10 min at 4°C to collect the supernatant, which was placed in a new 1.5 mL RNase-free centrifuge tube. After adding 200 μL of chloroform to the supernatant, thorough mixing, and centrifugation at 12,000 × *g* for 10 min at 4°C, the supernatant was collected. Then, after adding 500 μL of isopropanol to the supernatant, the resulting solution was mixed well and placed on ice for 10 min to precipitate the RNA. After centrifugation at 12,000 × *g* for 15 min at 4°C, RNA precipitates were collected, washed once with 70% ethanol, and dried on a clean bench.

cDNA was synthesized with a Smart cDNA synthesis kit (Takara, Dalian, China) with primers SMART F and Oligo anchor R (Table 1). A mixture of total RNA (5 μg of RNA in 9 μL of RNase-free water) and SMART F and Oligo anchor R primers (1 μL each) (Table 1) was incubated at 70°C for 5 min before placing in an ice bath. Then, 11 μL of the mixture was mixed with 1 μL of Power M-MLV (Bioteke, Beijing, China), 4 μL of 5 × first-strand cDNA buffer, 0.5 μL of RNase Inhibitor (Vazyme, Nanjing, China), 2 μL of dNTP mix solution (GENERAY, Shanghai, China), and 1.5 μL of RNase free water (total volume = 20 μL). cDNA was synthesized at 42°C for 1 h. At the end of cDNA synthesis, the reaction system was incubated at 70°C for 10 min to end the reaction. The obtained cDNA was used for subsequent experiments.

**Table 1:** Primers used in the present study.

**Primer**	**Sequence (5^**′**^-3^**′**^)**
**cDNA cloning and expression pattern analysis**	
*Mj*RPS27 RTF	CCTGGCTGCTTCAAGATTTC
*Mj*RPS27 RTR SMART F	GGTGACTTTCCGCAACATTA TACGGCTGCGAGAAGACGACAGAAGGG
Oligo anchor R	GACCACGCGTATCGATGTCGACT16(A/C/G)
**Reference gene**	
EF1α RTF	GGATTGCCACACCGCTCACA
EF1α RTR	CACAGCCACCGTTTGCTTCAT
**qPCR (WSSV envelope protein)**	
VP28RTF	AGCTCCAACACCTCCTCCTTCA
VP28RTR	TTACTCGGTCTCAGTGCCAGA
**qPCR (Transcription factors)**	
*Mj*Dorsal RTF	GCAATGCTGGTAACCTGGCTA
*Mj*Dorsal RTR	CTATGGATTTTGGTCAATACACTTT
*Mj*Relish RTF	CAGATAGATTCCTGTGCGTTGC
*Mj*Relish RTR	CGAGGTGGATTTCCGTTGTGT
**qPCR (Antimicrobial peptides)**	
*Mj*ALFB1 RTF	CGGTGGTGGCCCTGGTGGCACTCTTGG
*Mj*ALFB1 RTR	GACTGGCTGCGTGTGCTGGCTTCCCCTC
*Mj*ALFC1 RTF	CGCTTCAAGGGTCGGATGTG
*Mj*ALFC1 RTR	CGAGCCTCTTCCTCCGTGATG
*Mj*ALFC2 RTF	TCCTGGTGGTGGCAGTGGCT
*Mj*ALFC2 RTR	TGCGGGTCTCGGCTTCTCCT
*Mj*ALFD2 RTF	CGCAGGCTTATGGAGGAC
*Mj*ALFD2 RTR	AGGTGACAGTGCCGAGGA
*Mj*CrusI1 RTF	TGCTCAGAACTCCCTCCACC
*Mj*CrusI1 RTR	TTGAATCAGCCCATCGTCG
**RNAi**	
*Mj*RPS27 RNAiF	GCGTAATACGACTCACTATAGGATTTGATGACAGACCACTTC
*Mj*RPS27 RNAiR	GCGTAATACGACTCACTATAGGATCCATTTGCCACTTTAC
GFP RNAiF	GCGTAATACGACTCACTATAGGTGGTCCCAATTCTCGTGGAAC
GFP RNAiR	GCGTAATACGACTCACTATAGGCTTGAAGTTGACCTTGATGCC
**Recombinant expression**	
*Mj*RPS27 exF	CGCGGATCCATGCCTCTCGCAAAAGATTT
*Mj*RPS27 exR	CCGCTCGAGTTAGTTCTGCTTCCTTCTGA

### cDNA Cloning and Phylogenetic Analysis

The sequence of *MjRPS27* was obtained from the hemocyte transcriptome sequencing of *M*. *japonicus*. The sequence was amplified by RT-PCR with primers *Mj*RPS27 exF and exR (Table 1) and confirmed by resequencing. The confirmed sequence was analyzed at the blastx website (https://www.ncbi.nlm.nih.gov/), and its protein sequence was predicted with ExPASy-Traslate tool (https://web.expasy.org/translate/). The obtained protein sequence was analyzed with the ExPASy-Compute pI/Mw tool (https://web.expasy.org/compute_pi/). GENEDOC and MEGA5 were used for sequence alignment and phylogenetic-tree analysis, respectively.

### Tissue Distribution and Expression-Pattern Analysis

The tissue distribution of *MjRPS27* in shrimp was detected using semiquantitative RT-PCR with primers *Mj*RPS27 RTF and *Mj*RPS27 RTR (Table 1). The PCR profile was as follows: 94°C for 3 min, 35 cycles of 94°C for 15 s, 56°C for 20 s, 72°C for 20 s, and 72°C for 10 min. *EF1*α was used as an internal control. The DNA fragment obtained after PCR was detected by 1.5% agarose gel electrophoresis.

Quantitative real-time RCR (qPCR) was used to analyze the expression patterns of *MjRPS27* at different time points after WSSV challenge with primers *Mj*RPS27 RTF and *Mj*RPS27 RTR (Table 1). The PCR profile was as follows: 95°C for 10 min, 40 cycles of 95°C for 15 s, 60°C for 50 s, and read at 72°C for 2 s, and then a melt period from 65 to 95°C. PCR data were calculated using the 2^−ΔΔCT^ method and expressed as the mean ± SD. Student's *t*-test was used to analyze the significant differences among PCR data, and significant difference was accepted at *p* < 0.05.

### RNA Interference Assay

Double-stranded RNA synthesis and RNA Interference (RNAi) assay were both conducted as in our previous report (32). In a typical procedure, the software Primer Premier 5 was used to design the RNA interference primers of *MjRPS27* (Table 1). Simultaneously, GFP RNAiF and RNAiR were used to amplify the *dsGFP* fragment as a control (Table 1). First, a partial *MjPRS27* cDNA fragment was amplified by PCR with primers (*Mj*RPS27 RNAiF and *Mj*RPS27 RNAiR) linked to the T7 promoter sequence (Table 1) using for the template for dsRNA synthesis. The PCR profile for the template amplification was 94°C for 3 min, 35 cycles at 94°C for 30 s, 58°C for 30 s, 72°C for 40 s, and 72°C for 10 min. The PCR product was extracted using phenol–chloroform and was used as a template to synthesize dsRNA with an *in vitro* T7 Transcription Kit (Takara Bio, Dalian, China). The synthesis procedure of dsRNA was performed following manufacturer's instruction. The synthesized dsRNA was first extracted with phenol–chloroform, and the concentration was detected using a micro-spectrophotometer K5500 (K.O., China).

To test whether the expression of *MjRPS27* could be suppressed, we injected different amounts of dsRNA (40, 80, and 100 μg) into shrimp at the penultimate somite, and an equal amount of GFP dsRNA injection was used as the control. At 48 h post injection, the *MjRPS27* expression in gills and intestine was analyzed by qPCR to confirm the RNA interference (RNAi) efficiency. At least three shrimp were used for testing the RNAi efficiency.

After validating that *MjRPS27* expression could be silenced by the *dsRNA* injection, the RNAi assay was performed. Shrimp were randomly divided into two groups (30 individuals for each group). *DsRNA* (80 μg) was intramuscularly injected at the penultimate somite of shrimp, and the same amount of *dsGFP* was injected as a control. After 48 h of *dsRNA* injection, the WSSV (5 × 10^7^ copies/shrimp) were injected into shrimp of the two groups at the penultimate segment of shrimp with a microsyringe. WSSV replication in gills and intestines was analyzed by qPCR (using *VP28* expression as an indicator) 24 h post-WSSV injection using the primers VP28 RTF and VP28 RTR.

### Recombinant Expression and Purification of *Mj*RPS27

*Mj*RPS27 exF and *Mj*RPS27 exR (Table 1) were used to amplify *Mj*RPS27 by RT-PCR. The PCR product and empty plasmid pGEX4T-2 (GE Healthcare) were digested by two restriction endonucleases, *Bam*HI and *Xho*I (Thermo Scientific), at 37°C for 1 h (*MjRPS27*) and 37°C for 0.5 h (vector pGEX4T-2). The obtained fragments and the pGEX4T-2 plasmids ligated with T4 DNA ligase (Thermo Fisher) to construct the recombinant plasmid pGEX4T-2/*Mj*RPS27. The constructed recombinant plasmid was then transformed into *E. coli* DH5α cells, cultured at 37°C overnight. The recombinant plasmid, purified from the *E. coli* DH5α, was transformed into *E. coli* Rosetta cells, and *Mj*RPS27 expression was induced with β-d-1-thiogalactopyranoside (IPTG; Sangon, Shanghai, China) at a final concentration of 0.5 mM at 37°C. Rosetta bacteria were collected and disrupted with ultrasonic waves. The crushed bacterial solution was centrifuged at 12,000 × *g* for 10 min at 4°C, and the supernatant and precipitate were collected and analyzed by sodium dodecylsulfate-polyacrylamide gel electrophoresis (SDS-PAGE). The recombinant protein was purified by GST-resin chromatography (GenScript, Nanjing, China) following the manufacturer's instructions.

### Pulldown Assay

To analyze the interaction of *Mj*RPS27 with WSSV envelope proteins, a pulldown assay was performed. *Mj*RPS27 with GST tag, GST tag protein, and four types of WSSV envelope proteins (VP19, VP24, VP26, and VP28) with His tag were expressed in *E. coli* using our previously constructed plasmid (33). Recombinant *Mj*RPS27 and different envelope proteins (100 μg) were mixed and incubated at 4°C overnight. Then, 100 μL of glutathione-Sepharose was added to the mixture and incubated at 4°C for 40 min. The mixture (resin and binding proteins) was washed six times with PBS by centrifugation at 500 × *g* for 3 min to remove the unbound proteins. The interacting proteins were eluted by 50 μL of GST elution buffer (10 mM glutathione, pH 8.0), and the eluted solution was subjected to Western blot analysis.

### Western Blot Assay

Previously obtained samples were mixed with 25 μL of sodium dodecylsulfate loading buffer and subjected to 12.5% SDS-PAGE analysis following the Laemmli method (34). The proteins in the SDS-PAGE gel were electrotransferred onto a nitrocellulose membrane (pore size = 0.45 μm). Non-fat dry milk was dissolved in TBS (10 mM Tris-HCl and 150 mM NaCl; pH 8.0), and the 3% non-fat dry milk solution was placed on the blocking membrane. After incubation at room temperature for 1 h, the blocking solution was discarded and the primary antibody (against His tag or GST tag; Zhongshan, Beijing, China) (1:10,000 diluted in blocking solution) was added. After overnight incubation at 4°C, the nitrocellulose membrane was washed with TBST (TBS plus 0.1% Tween-20) three times for 10 min each time. The membrane was then incubated for 3 h in horseradish-labeled goat anti-mouse IgG (Zhongshan, Beijing, China) (1:10,000 in blocking solution), washed three times for 10 min in TBST, and washed in TBS for 5 min. The nitrocellulose membrane was developed by horseradish peroxidase method by adding 1 mL of 4-chloro-1-naphthol in methanol (6 mg/mL) to 9 mL of TBS and 6 μL of H_2_O_2_ and reacting for 10 min.

### Recombinant Expression of *Mj*RPS27 With Cell-Penetrating TAT Peptide

To ensure the entry of recombinant *Mj*RPS27 into the cells, we fused the sequence encoding the cell-penetrating TAT peptide (TATGGAGAGGAAGAAGCGGAGACAGCGACGAAGA) (35) with *Mj*RPS27 and then constructed pET30a–TAT–*Mj*RPS27 to express the TAT–*Mj*RPS27 fusion protein in *E. coli*. The protein was purified by Ni-resin chromatography (GenScript, Nanjing, China) and used for the overexpression assay in shrimp.

### Immunocytochemistry Assay

To detect whether the recombinant protein could enter shrimp cells, healthy shrimp were divided into two groups (10 individuals for each group), and the TAT–*Mj*RPS27 protein was injected into the shrimp (10 μg/shrimp). The same amount of TAT-His tag protein and BSA was also injected as a control. The hemolymph was extracted using sterile syringes with anticoagulant and a 4% paraformaldehyde solution. Hemocytes were collected by centrifuging at 800 × g for 6 min at 4°C. Afterwards, the hemocytes were washed with anticoagulant and a 4% paraformaldehyde solution. The hemocytes were dropped onto polylysine-treated slides and allowed to stand for 1 h in a wet box. Triton-X100 (0.2%) was added to the slides for 5 min and washed with PBS six times (5 min each time). After blocking with 3% BSA (in PBS) at 37°C for 30 min, the primary antibody against His tag (1:1,000 dilution with 3% BSA; Zhongshan, Beijing, China) was added to the glass slides and incubated at 37°C overnight. After washing with PBS six times, the glass slides were blocked with 3% BSA (in PBS) at 37°C for 30 min. Fluorescein isothiocyanate-conjugated secondary antibody was then added to the slides in darkness at 37°C for 1 h. After washing with PBS six times, the glass slides were incubated with 4′,6-diamidino-2-phenylindole (DAPI) (1 μg/mL) for 10 min in darkness and washed six times to remove excess DAPI. Finally, the glass slides were observed under a fluorescence microscope (Olympus DP71).

The fluorescence immunocytochemical assay was also used to detect the nuclear translocation of *Mj*Dorsal and *Mj*Relish after *Mj*RPS27 knockdown. The anti-Dorsal and anti-Relish sera used in the assay were prepared in our laboratory (36). The healthy shrimp were divided into two groups and 10 shrimp were used in each group. *DsMjRPS27* and *dsGFP* were firstly injected into shrimp, WSSV was then injected 48 h post *dsMjRPS27* injection. After 2 h of WSSV injection, hemocytes were collected for immunocytochemical analysis. *dsGFP* and WSSV injection shrimp served as controls.

The immunocytochemical assay was performed as described above. The anti-*Mj*Dorsal or anti-*Mj*Relish (1:400 in 3% bovine serum albumin) was used as primary antibody, and the Alexa Fluor 488-conjugated antibody to rabbit (1:1,000 ratio, diluted in 3% BSA) was used as the second antibody. The number of cells of *Mj*Dorsal or *Mj*Relish into the nucleus or not into nucleus in different fields was observed under a microscope (Shinjuku-ku, Tokyo, Japan), and about 100 cells were counted. The experiment was repeated three times, the *dsGFP* injection was used as a control. A student's *t*-test was used to analyze difference significant among these data, and significant difference was accepted at *p* < 0.05.

### Overexpression Assay

To further confirm the function *Mj*RPS27 in shrimp, a type of *Mj*RPS27 “overexpression” was performed by TAT–*Mj*RPS27 injection following previous report (37, 38). Shrimp were divided into two groups (10 individual each). The TAT–*Mj*RPS27 recombinant protein was mixed with WSSV and immediately injected into shrimp (10 μg/shrimp), and a TAT-His tag plus a WSSV injection was used as a control. The mixture was intramuscularly injected into shrimp at the penultimate segment with a microsyringe. Hemocytes and gills were collected at 24 and 48 h post injection, and WSSV replication was analyzed by qPCR using VP28 expression as an indicator.

### *Mj*Dorsal, *Mj*Relish, and Related AMP Detection After Knockdown of *MjRPS27* by RNAi

To determine whether the immune function of *Mj*RPS27 was related to NF-κB pathways, we detected the expression of *Mj*Dorsal, *Mj*Relish, and related antimicrobial peptides of NF-κB pathways (RT-PCR primers in Table 1) in the intestine after *Mj*RPS27 knockdown by RNAi. The qPCR data analysis method was the same as that which was mentioned before.

### Assessment of Survival Rates

To further confirm the role of *Mj*RPS27 in the WSSV infection of *M. japonicus*, we performed a survival assay for the injection of recombinant protein (a type of overexpression). We initially confirmed that the constructed recombinant protein with a cell-penetrating TAT peptide can enter the cell. Shrimps were randomly divided into two groups with 40 animals per group. In the first one, the mixture of TAT–*Mj*RPS27 (50 μg/shrimp) and WSSV (5 × 10^7^ copies per shrimp) was intramuscularly injected into the penultimate segment of the shrimp; the same amount of TAT-His tag purified from the empty pET30a–TAT vector together with WSSV was injected into the second group. The number of dead shrimps was counted every 12 h after injection, and the survival rate of the shrimp was calculated and analyzed using GraphPad Prism software.

### Statistical Analysis

Data are represented as the results of at least three independent experiments. Student's *t*-tests were used to calculate significance at *p* < 0.05 (^*^), *p* < 0.01(^**^), and *p* < 0.001(^***^). Some data (the nuclear translocation rates of *Mj*Dorsal and *Mj*Relish) were subjected to one-way ANOVA with a Scheffe test from triplicate assays. Significant differences (*p* < 0.05) are represented by different letters.

## Results

### *MjRPS27* Was Upregulated in Shrimp Challenged by WSSV

*MjRPS27* consists of 375 bp with a 252 bp opening reading frame, which encodes 84 amino acids (GenBank MN385248, Figure S1). The theoretical values of the isoelectric point and molecular weight are 9.47 and 9222.93, respectively. The sequence alignment analysis (Figure S2A) and phylogenetic-tree analysis (Figure S2B) showed that RPS27 exhibited high sequence conservation in different organisms, and that *Mj*RPS27's sequence was highly similar to that of *Litopenaeus vannamei* and *Limulus polyphemus*.

**Figure 1:**
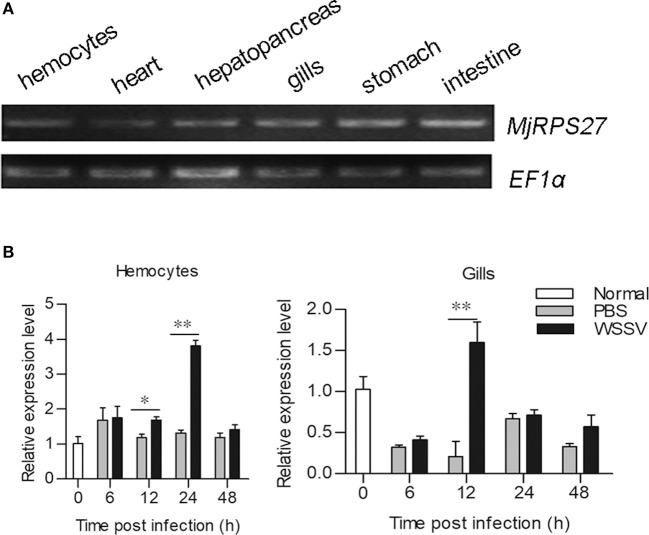
Tissue distribution and expression pattern of *Mj*RPS27. **(A)** Tissue distribution of *Mj*RPS27 mRNA. **(B)** Expression pattern of *Mj*RPS27 in hemocytes and gills of shrimp challenged by WSSV as analyzed by qPCR. PBS injection shrimp served as a control. At least three shrimp were used for hemocytes and tissue collection at different time points. Significance was compared between the infected group and the same time point by *t*-test analysis, and significant difference was accepted at *p < 0.05; **p < 0.01.

The tissue distribution of *MjRPS27* in shrimp was analyzed by RT-PCR, and results showed that *MjRPS27* was expressed in all tested tissues (Figure 1A). To determine whether *MjRPS27* responded to WSSV infection, the expression pattern was analyzed by qPCR. The expression of *MjRPS27* was found to increase significantly in hemocytes 12 and 24 h after WSSV injection. *MjRPS27* was also upregulated in the gills of shrimp challenged with WSSV (Figure 1B). These results suggested that *Mj*RPS27 was involved in viral infection in shrimp.

### WSSV Replication Increased in *MjRPS27*-Knockdown Shrimp

**Figure 2:**
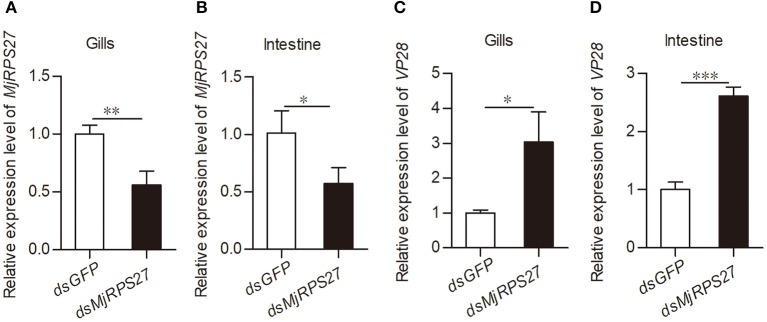
*VP28* expression was increased after knockdown of *MjRPS27*. **(A,B)** The RNAi efficiency of *MjRPS27* was analyzed by qPCR 48 h post-dsRNA injection. The same amount of *dsGFP* injection served as a control. **(C,D)** After knockdown of *MjRPS27*, the shrimp were challenged with WSSV (5 × 10^7^ copies per shrimp), and the expression levels of *VP28* were analyzed by qPCR 24 h post-WSSV challenge. Compared with the controls (*dsGFP* injection), the mRNA expression level of *VP28* significantly increased. *p < 0.05; **p < 0.01; ***p < 0.001.

To examine the function of *Mj*RPS27 in the WSSV infection of shrimp, we conducted an RNA interference experiment and followed the WSSV infection. After 48 h of dsRNA injection, the mRNA expression of *MjRPS27* was detected by qPCR. *MjRPS27* expression was successfully knocked down in the gills (Figure 2A) and intestine (Figure 2B). Then, the *MjRPS27* knockdown shrimp were challenged with WSSV (5 × 10^7^ copies per shrimp). The expression level of WSSV envelope protein 28 (VP28) was analyzed by qPCR after 24 h. Compared with the control (*dsGFP* injection), the expression level of *VP28* significantly increased (Figures 2C,D). To confirm the *MjRPS27* RNAi was a specific knockdown of the gene, we analyzed expressions of other genes in hemocytes and the intestine of the *MjRPS27*-silenced shrimp by qPCR (Table S1), including a related gene, ribosomal protein S28 (*MjRPS28*), a transcription factor gene *MjFOXO*, and other unrelated genes, such as a G protein-coupled receptor with methuselah domain (*MjMthGPCR*), a serine/threonine-protein kinase (*MjAKT*), and two small GTPases (*MjRab5* and *MjRab7*). The results showed that the expression of all above genes was not altered compared with controls in hemocytes and the intestine (Figure S3), suggesting that there was no non-specific silencing of the gene after injection of dsRNA of *MjRPS27*. Based on the above results, we can speculate that *Mj*RPS27 can inhibit WSSV proliferation in shrimp.

### WSSV Replication Decreased in *Mj*RPS27-Overexpressed Shrimp

**Figure 3:**
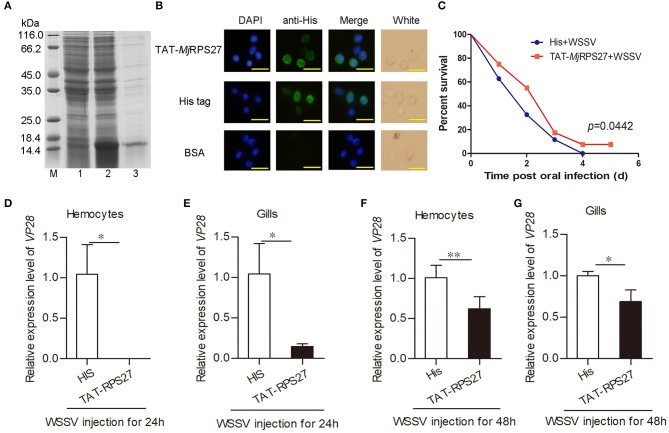
TAT–*Mj*RPS27 overexpression inhibited WSSV proliferation. **(A)** Expression and purification of recombinant of TAT-*Mj*RPS27. Lane M, protein marker; lane 1, total proteins from *E. coli*; lane 2, total proteins from *E. coli* after induced by IPTG; and lane 3, purified recombinant protein. **(B)** Immunocytochemistry to detect recombinant protein in hemocytes. Top panel, recombinant protein (TAT-*Mj*RPS27) injection (10 μg/shrimp); middle, His tag protein injection; bottom, BSA injection. Bars = 20 μm. **(C)** Survival rate of shrimp injected with TAT-*Mj*RPS27 (50 μg/shrimp); His tag protein injection served as a control. **(D)** *VP28* expression in hemocytes of TAT-*Mj*RPS27-WSSV-injected shrimp analyzed by qPCR at 24 h post-injection, His-WSSV-injected shrimp served as a control. **(E)** *VP28* expression in gills of TAT-*Mj*RPS27-WSSV-injected shrimp analyzed by qPCR at 24 h post injection, His-WSSV-injected shrimp served as a control. **(F,G)** *VP28* expression in hemocytes **(F)** and Gills **(G)** of TAT-*Mj*RPS27 and WSSV-injected shrimp was analyzed by qPCR 48 h post injection. His-WSSV-injected shrimp served as a control. PCR data were calculated using the 2^−ΔΔCT^ method and expressed as the mean ± SD. Student's *t*-test was used to analyze significant differences among PCR data, and significant difference was accepted at *p < 0.05; **p < 0.01.

To further confirm the function of *Mj*RPS27 in the antiviral immunity of shrimp, an “overexpression” assay was performed. The TAT–*Mj*RPS27 fusion protein was expressed and purified (Figure 3A), and then healthy shrimp were injected with the recombinant protein. To determine whether the recombinant protein can enter cells, an immunocytochemistry assay was performed using anti-His tag as the primary antibody. As shown in Figure 3B, TAT–*Mj*RPS27 was detected in hemocytes 1 h post injection. After observing that the recombinant protein can enter hemocytes, the overexpression assay was performed and the WSSV replication and survival rate of shrimp were analyzed. We firstly mixed TAT–*Mj*RPS27 protein (50 μg/shrimp) and WSSV (5 × 10^7^ copies/shrimp) together and immediately injected this into the shrimp. Compared with the control (TAT-His tag protein and WSSV injection), the shrimp survival rate significantly increased (Figure 3C). *VP28* expression in hemocytes and gills was also analyzed by qPCR 24 and 48 h post injection. Results showed that *VP28* expression was significantly inhibited in hemocytes and gills at 24 and 48 h post injection (Figures 3D–G). All these findings indicated that *Mj*RPS27 can inhibit the replication of WSSV in *M. japonicus*.

### *Mj*RPS27 Involvement in the Regulation of NF-κB Pathways

**Figure 4:**
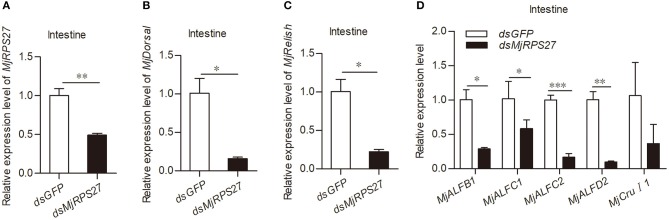
*Mj*RPS27 was involved in the activation of the Toll and IMD pathways. **(A)** The RNA interference efficiency of *Mj*RPS27 was analyzed by qPCR 48 h after *dsMjRPS27* injection. The same amount of *dsGFP* was injected as a control. **(B)** After knockdown of *Mj*RPS27, the expression level of *Mj*Dorsal was analyzed by qPCR. The mRNA expression level of *MjDorsal* was significantly downregulated compared with the control (*dsGFP* injection). **(C)** After knockdown of *MjRPS27*, the expression level of *Mj*Relish was analyzed by qPCR. The mRNA expression level of *MjRelish* was significantly downregulated compared with the control (*dsGFP* injection). **(D)** After knockdown of *Mj*RPS27, the expression levels of AMPs (*Mj*ALFB1, *Mj*ALFC1, *Mj*ALFC2, *Mj*ALFD2, and *Mj*CruI 1) were analyzed by qPCR. The expression levels of AMPs, except *MjCruI 1*, were significantly downregulated compared with the control (injected *dsGFP*). *p < 0.05; **p < 0.01; ***p < 0.001.

Previous studies have shown that RPS27 is related to the activation of NF-κB pathways in cancer cells (23). In our previous studies, we found that the Toll pathway in shrimp can regulate the expression of several antimicrobial peptides (AMPs), such as anti-lipopolysaccharide factor C2 (ALF-C2) and Crustin I-1 (CruI-1). The IMD pathway can regulate the expression of ALF-B1, ALF-C1, and ALF-D2 (25, 39). Accordingly, we evaluated the expression of Dorsal (transcription factor of the Toll pathway), Relish (transcription factor of the IMD pathway), and AMPs in shrimp after *Mj*RPS27 knockdown (Figure 4A). Compared with the control group, the expression levels of *Mj*Dorsal and *Mj*Relish decreased significantly (Figures 4B,C); the expression levels of *Mj*ALFB1, *Mj*ALFC1, *Mj*ALFC2, and *Mj*ALFD2 in the intestine also decreased significantly (Figure 4D). These results suggested that *Mj*RPS27 was involved in the regulation of the Toll and IMD pathways and that its antiviral function may be related to the upregulation of AMPs in shrimp.

### Decreased Nuclear Translocation of *Mj*Dorsal and *Mj*Relish After *MjRPS27* Knockdown

**Figure 5:**
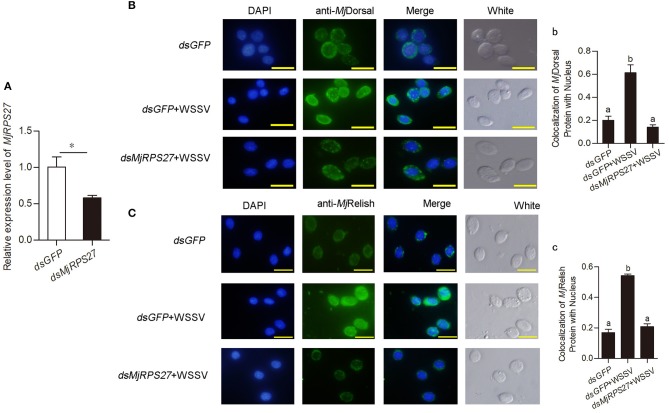
The nuclear translocation of *Mj*Dorsal and *Mj*Relish decreased after knockdown of *Mj*RPS27 in shrimp. **(A)** Efficiency of *Mj*RPS27 interference in gills; *dsGFP* injection served as a control. **(B)** Immunocytochemical analysis to detect the nucleus translocation of *Mj*Dorsal in hemocytes of shrimp after RNAi of *MjRPS27*; *dsGFP*-injected shrimp served as a control. Bars = 20 μm. (b) Statistics analysis of *Mj*Dorsal and nucleus co-localization in hemocytes. **(C)** Immunocytochemical analysis used to detect the nucleus translocation of *Mj*Relish in hemocytes of shrimp after RNAi of *MjRPS27*; *dsGFP*-injected shrimp served as a control. Bars = 20 μm. (c) Statistics analysis of the colocalization of *Mj*Relish with nucleus in hemocytes (details described in Materials and Methods). *p < 0.05.

Previous study found that knockdown of MPS-1/RPS27 inhibited NF-κB activity by reducing the phosphorylation of p65 and inhibiting NF-κB nuclear translocation in human gastric cells (23). We used antibodies against *Mj*Dorsal and *Mj*Relish prepared in our laboratory to determine whether *Mj*RPS27 affected the *Mj*Dorsal and *Mj*Relish entry into the nucleus. After knockdown of *Mj*RPS27 and WSSV injection, *Mj*Dorsal and *Mj*Relish in hemocytes were detected by fluorescence immunocytochemical assay, and the same amount of *dsGFP* was injected as a control. Results showed that, after *MjRPS27* knockdown (Figure 5A), the entry rate of *Mj*Dorsal and *Mj*Relish into the nucleus decreased significantly (Figures 5Bb,Cc). These results indicated that *Mj*RPS27 can regulate the nuclear translocation of *Mj*Dorsal and *Mj*Relish and subsequently regulate the expression of AMPs to prevent viral proliferation in shrimp infected by WSSV.

### Expression and Purification of *Mj*RPS27 and WSSV Envelope Proteins

**Figure 6:**
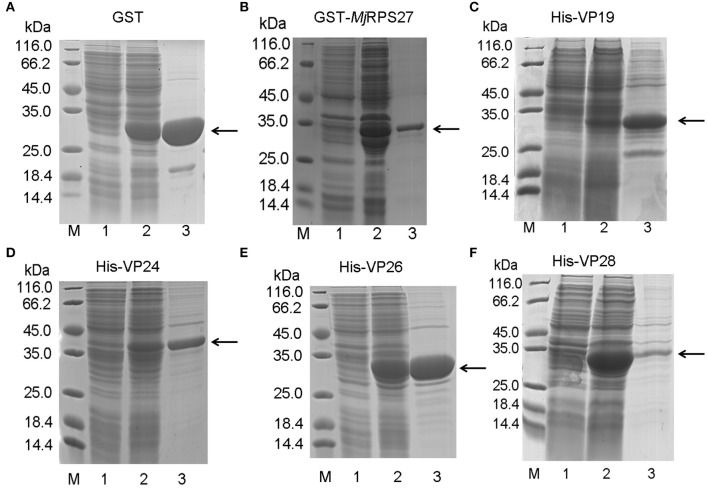
Recombinant expression and purification of *Mj*RPS27 and WSSV envelope proteins. **(A)** Recombinant expression and purification of GST tag protein. Lane M, protein marker; lane 1, total proteins from *E. coli*; lane 2, total proteins from *E. coli* after induction by IPTG; and lane 3, purified GST protein. **(B)** Recombinant expression and purification of *Mj*RPS27. Lane M, protein marker; lane 1, total proteins from *E. coli* with pGEX4T-2/*Mj*RPS27; lane 2, total proteins from *E. coli* after induction by IPTG; and lane 3, purified *Mj*RPS27. **(C)** Recombinant expression and purification of WSSV VP19. Lane M, protein marker; lane 1, total proteins from *E. coli* with pET-32a(+)/VP19; lane 2, total proteins from *E. coli* after induction by IPTG; and lane 3, purified recombinant protein. **(D)** Recombinant expression and purification of WSSV VP24. Lane M, protein marker; lane 1, total proteins from *E. coli* with pET-32a(+)/VP24; lane 2, total proteins from *E. coli* after induction by IPTG; and lane 3, purified VP24. **(E)** Recombinant expression and purification of VP26. Lane M, protein marker; lane 1, total proteins from *E. coli* with pET-32a(+)/VP26; lane 2, total proteins from *E. coli* after induction by IPTG; and lane 3, purified VP26. **(F)** Recombinant expression and purification of WSSV VP28. Lane M, protein marker; lane 1, total proteins from *E. coli* with pET-30a(+)/VP28; lane 2, total proteins from *E. coli* after induction by IPTG; and lane 3, purified VP28. The arrows indicated the purified proteins.

To study the function and possible mechanism of *Mj*RPS27 in shrimp immunity, we also analyzed *Mj*RPS27 interaction with envelope proteins of WSSV. We initially expressed the GST-tagged *Mj*RPS27 protein and His-tagged envelope proteins of WSSV for pulldown analysis. Figure 6 shows the purified recombinant proteins, including the GST tag protein expressed in *E. coli* (Figure 6A), purified GST-tagged *Mj*RPS27 expressed in *E. coli* with pGEX4T-2/*Mj*RPS27 (Figure 6B), and four types of His-tagged envelope proteins of WSSV expressed in *E. coli* with pET-32a(+)/VP19, pET-32a(+)/VP24, pET-32a(+)/VP26, and pET-30a(+)/VP28 (Figures 6C–F).

### *Mj*RPS27 Interaction With VP19, VP24, and VP28 of WSSV

**Figure 7:**
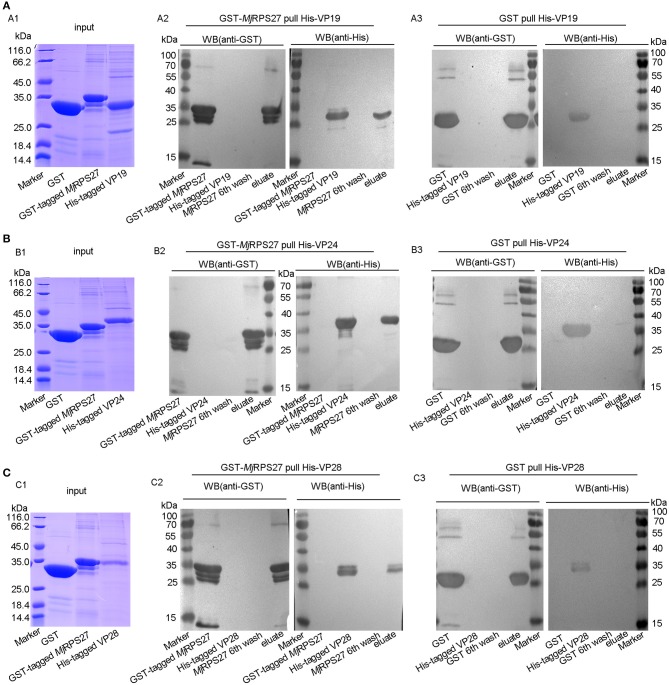
*Mj*RPS27 interacted with the WSSV envelope proteins, VP19, VP24, and VP28. **(A)** The interaction of *Mj*RPS27 with VP19 was analyzed by GST pulldown. (A1) Input proteins (GST protein, GST-tagged *Mj*RPS27, and His-tagged VP19) were analyzed by SDS-PAGE. (A2) GST-*Mj*RPS27 pulldown His-VP19 was analyzed by Western blot: left panel, proteins used in the Pulldown assay were initially separated by SDS-PAGE, electrotransferred onto a nitrocellulose membrane, and analyzed by Western blot using anti-GST as primary antibody; right panel, SDS-PAGE-separated proteins were transferred onto the nitrocellulose membrane and analyzed by Western blot using anti-His as primary antibody. (A3) GST pulldown His-VP19 analyzed by Western blot: left panel, Western blot with anti-GST; right panel, Western blot using anti-His as primary antibody (control). **(B)** The interaction of *Mj*RPS27 with VP24 was analyzed by GST pulldown. (B1) Input proteins (GST protein, GST-tagged *Mj*RPS27 and His-tagged VP24) were analyzed by SDS-PAGE. (B2) GST-*Mj*RPS27 pulldown His-VP24 was analyzed by Western blot: left panel, Western blot with anti-GST; right panel, Western blot analysis using anti-His as primary antibody. (B3) GST pulldown His-VP24 was analyzed by Western blot: left panel, Western blot with anti-GST; right panel, Western blot using anti-His as primary antibody (control). **(C)** The interaction of *Mj*RPS27 with VP28 was analyzed by GST pulldown. (C1) Input proteins (GST protein, GST-tagged *Mj*RPS27, and His-tagged VP28) were analyzed by SDS-PAGE. (C2), GST-*Mj*RPS27 pulldown His-VP28 analysis: left panel, Western blot analysis with anti-GST; right panel, Western blot analysis using anti-His as primary antibody. (C3) GST pulldown His-VP28 analysis: left panel, Western blot analysis with anti-GST; right panel, Western blot analysis using anti-His as primary antibody (control).

To study the possible mechanism of *Mj*RPS27 activity against WSSV in shrimp, the interaction of the protein with WSSV was analyzed. GST pulldown was carried out using purified *Mj*RPS27 and WSSV envelope proteins. Given that the molecular weight of *Mj*RPS27 with GST tag protein is similar to those of WSSV envelope proteins, we detected the interactions by Western blot assay. The proteins (GST, GST-tagged *Mj*RPS27, and His-tagged VP19) used for the interaction analysis are shown in Figure 7A1. We observed a band in the elution lanes detected by anti-GST (Figure 7A2, left panel) and anti-His (Figure 7A2, right panel); however, in the control (Figure 7A3), only a band in the elution lane was detected by anti-GST (Figure 7A3, left panel), and no band in the elution lane was detected by anti-His (Figure 7A3, right panel). These results suggested that *Mj*RPS27 interacted with VP19, but GST protein cannot interact with VP19. The same results were obtained in the interaction analysis of *Mj*RPS27 with VP24 (Figure 7B) and *Mj*RPS27 with VP28 (Figure 7C). All these results suggest that *Mj*RPS27 can interact with VP19, VP24, and VP28 but not with VP26 (Figure S4). Based on these findings, we can conclude that *Mj*RPS27 interacted with VP19, VP24, and VP28 proteins, indicating that *Mj*RPS27 may inhibit the proliferation of WSSV by interacting with the envelope proteins to prevent WSSV invasion and assembly. We could also assume that *Mj*RPS27 recognized infected WSSV through interaction with envelope proteins and activated the NF-κB pathway to induce expression of AMPs.

## Discussion

Several studies have implicated SEPs in response to infection and innate immunity, but the mechanisms were unclear for most of them. In the present study, we found that *Mj*RPS27 was upregulated after WSSV stimulation, suggesting that *Mj*RPS27 may participate in the immune response against WSSV in *M. japonicus*. Further mechanism study shows that *Mj*RPS27 interacts with WSSV envelope proteins and inhibits WSSV infection by activating the NF-κB pathways to induce the expression of AMPs in shrimp.

In the present study, we knocked down the expression of *Mj*RPS27 and detected the expression of VP28 after WSSV challenge. We found that the expression of VP28 was significantly increased. To confirm the function of *Mj*RPS27, we constructed a type of overexpression assay using the recombinant *Mj*RPS27 carrying a cell-penetrating TAT peptide to enhance the cellular uptake of the protein. Results showed that the expression of VP28 in shrimp significantly decreased and that the survival rate of shrimp increased significantly compared with that of the control group. Overall, the findings suggested that *Mj*RPS27 can inhibit WSSV infection in shrimp.

Dorsal is a known NF-κB transcription factor in the classical Toll pathway. In *Litopenaeus vannamei*, the silence of Dorsal leads to a decline in the expression of a specific group of AMPs, such as the anti-lipopolysaccharide factor (ALF) and lysozyme (LYZ) family. Meanwhile, ALF1 and LYZ1 have been shown to interact with several WSSV structural proteins to inhibit viral infection (40). In other studies, the Toll and IMD pathways in shrimp are activated and AMP are induced after WSSV infection. Some AMPs and other immune-related genes such as ALF, penaeidin, and PMAV (an antiviral gene from *Penaeus monodon* encoding a protein with a C-type lectin-like domain) have direct antiviral activity (41–44). Accordingly, after *Mj*RPS27 knockdown, we detected the expression of AMPs and the nuclear translocation of *Mj*Dorsal and *Mj*Relish. We found that the nuclear translocation of *Mj*Dorsal and *Mj*Relish were significantly reduced (Figure 5) and that the expression of AMPs was markedly downregulated (Figure 4). All the results suggested that *Mj*RPS27 can activate the NF-κB pathways and induce the expression of AMPs, thereby playing an antiviral role in shrimp. The similar results were reported in human gastric cancer cells. Knockdown of MSP-1/RPS27 can inhibit NF-κB activity by reducing phosphorylation of p65 and IκB, inhibiting NF-κB nuclear translocation, and downregulating its DNA binding activity (23).

How does the RPS27 activate the NF-κB pathway? It is reported that some ribosomal proteins can directly regulate gene transcription or modulate transcriptional factors (23). Ribosomal proteins usually contain sequences such as zinc finger motifs, which enables them to interact instantaneously or stably with DNA and RNA. For example, ribosomal protein S3 (RPS3) can interact with NF-κB, with p65 forming as a subunit of a p65-dimer and p65-p50 isodimer DNA binding complex, which enhances DNA binding by stabilizing the binding of Rel subunit to some homologous sites. RPS3 knockout impairs the activity of NF-κB and the transcription of its target gene (45). Knocking down MPS-1 (RPS27) in gastric cancer cells can affect the activity of NF-κB. It is speculated that MPS-1, like RPS3, can directly or indirectly regulate the activity of NF-κB by binding to the target DNA of NF-κB and stabilizing the protein–DNA complex as a transcriptional co-regulator with zinc finger domain (23). The knockout of MPS-1 proteins reduces the stability of complexes and their interaction with target genes, thereby reducing the activity of NF-κB (23). We speculated that *Mj*RPS27 may directly or indirectly regulate the activity of *Mj*Dorsal and *Mj*Relish by binding their target DNA and stabilizing protein–DNA complexes. The detailed mechanism of RPS27 activating the NF-κB pathway needs further investigation.

In previous studies, we have found that some proteins can inhibit the replication of WSSV by binding with WSSV proteins. For example, prohibitin can inhibit the proliferation of WSSV by binding with VP28, VP26, and VP24 in crayfish (46). Some envelope proteins of WSSV can also be involved in the process of virus infection. For example, the major envelope protein VP28 reportedly plays a key role in the initial stage of systemic WSSV infection in shrimp (47). Moreover, WSSV VP28, as an attachment protein, has been found to play an important role in the process of infection, such as through binding the virus to shrimp cells and helping them enter the cytoplasm (48). The interaction between *Mj*RPS27 and WSSV envelope proteins (VP19, VP24, and VP28) was examined by GST pulldown. Results showed that *Mj*RPS27 can bind to VP19, VP24, and VP28, suggesting that *Mj*RPS27 can also inhibit the invasion, assembly, and proliferation of envelope proteins by binding to WSSV envelope proteins.

In conclusion, our study showed that a sORF-encoded protein, *Mj*RPS27, participated in the innate immune process of shrimp infected by WSSV. *Mj*RPS27 played an antiviral role by binding with WSSV envelope proteins and activating the NF-κB pathway. Our research further enriched knowledge on invertebrate antiviral innate immunity and the functional diversity of RPS27.

## Data Availability Statement

The datasets generated for this study can be found in Genbank under the accession number MN385248. The other data supporting the conclusions of this manuscript will be made available by the authors, without undue reservation, to any qualified researcher.

## Author Contributions

J-XW and X-FZ supervised the overall project and designed the experiments. M-QD performed the experiments, analyzed data. J-DX helped to perform experiments and analyzed data. CL performed RNA interference experiment and helped to analyze data.

### Conflict of Interest

The authors declare that the research was conducted in the absence of any commercial or financial relationships that could be construed as a potential conflict of interest.
