# Supplementary material for: RPS27, a sORF-Encoded Polypeptide, Functions Antivirally by Activating the NF-κB Pathway and Interacting With Viral Envelope Proteins in Shrimp
Source: Front Immunol. 2019 Dec 17;10:2763. doi: 10.3389/fimmu.2019.02763 (PMC6928191; doi:10.3389/fimmu.2019.02763)
Supplement: Supplementary file 1 [file Data_Sheet_1.pdf]

Supplementary information for

RPS27, a sORF-encoded polypeptide functions antivirally by activating NF-κB pathway and interacting with viral envelope proteins in shrimp

Meng-Qi Diao[1#], Cang Li[1], Ji-Dong Xu[1], Xiao-Fan Zhao[1], Jin-Xing Wang[1,2,*]

1. Shandong Provincial Key Laboratory of Animal Cells and Developmental Biology, School of Life Science, Shandong University, Qingdao 266237, Shandong, China.

2. State Key Laboratory of Microbial Technology, Shandong University, Qingdao, China; Laboratory for Marine Biology and Biotechnology, Qingdao National Laboratory for Marine Science and Technology, Qingdao 266237, Shandong, China.

Corresponding author:
Jin-Xing Wang
Email: jxwang@sdu.edu.cn

[#] Present address:
Shandong Academy of Pharmaceutical Sciences, Key Laboratory of Biopharmaceuticals, Engineering Laboratory of Polysaccharide drugs, National-Local Joint Engineering Laboratory of Polysaccharide drugs, Postdoctoral Scientific Research Workstation, Jinan, 250101, Shandong, China

Materials and methods

RNA Interference Assay for Analyzing Specific mRNA Silencing of *Mj*RPS27

Double-stranded *Mj*RPS27 synthesis and RNA Interference (RNAi) assays were conducted as in our previous report [1]. To test the specificity of *Mj*RPS27 knockdown in RNAi, we detected expression of several related or unrelated genes by qPCR after *dsMjRPS27* injection with related primers (Table S1). We selected the ribosomal protein S28 (*MjRPS28*) as a related gene; and a transcription factor gene, FOXO, and other unrelated genes, such as a G protein-coupled receptor with methuselah domain (*MjMthGPCR*), a serine/threonine-protein kinase (*MjAKT*) and two small GTPases (*MjRab5 and MjRab7*) for expression analysis in hemocytes and intestine.

Results:

1. The nucleotide sequence and predicted amino acid sequence of *Mj*RPS27.

```
1    ctcgaaatttccaaaacaaaatcaaaatgaaaaatcataacgtcgccatattaaccaatcg
61   ataaaatcaacacgcacatgcgctataacaaaccagaaaaataaaaacaacacaaaaaga
121  gcaccttaaagccccaaaacgtggcgtaaatcggcacctaacgagatctataaaggttgc
181  gcatcccgacgctactttgttactggaggacgaagtttagctcttttcggccgttctcgg
241  attaagatcaacatgcctctcgcaaaagatttacttcatccctcacctgctgaggagaag
1                   M  P  L  A  K  D  L  L  H  P  S  P  A  E  E  K
301  aagaaatgcaagctcaaacgccttgtgcagcacccaaactcctacttcatggatgttaag
16    K  K  C  K  L  K  R  L  V  Q  H  P  N  S  Y  F  M  D  V  K
361  tgccctggctgcttcaagatttccacagtttctcccacgcccagacagtagtagcatgt
36    C  P  G  C  F  K  I  S  T  V  F  S  H  A  Q  T  V  V  A  C
421  gtaggctgtgcaacagttctatgccagcctactggtgggaaggctaagcttacagatgga
56    V  G  C  A  T  V  L  C  Q  P  T  G  G  K  A  K  L  T  D  G
481  tgttcattcagaaggaagcagaactaagttgggagagaaaatgcacctcccagtgtagac
76    C  S  F  R  R  K  Q  N  -
541  taaatatcctgtcgctatgtaacctagacagcattaaagatcccactgagaggttgatat
601  ttgttttattacgttcactttctaaggaataaatagctttaatgttgcggaaagtcaccg
661  tgttagctttttgtcaaagttttgtactttttgaaaggaataaatgtctgcattgtagt
721  ggactttaattgtacaaaaggctgttggtaaggaaaagtgctgtaatgggaatatatatt
```

Figure S1. The nucleotide sequence and predicted amino acid sequence of *Mj*RPS27. The shaded part is the zinc finger domain.

2. Alignment and Phylogenetic analysis of RPS27

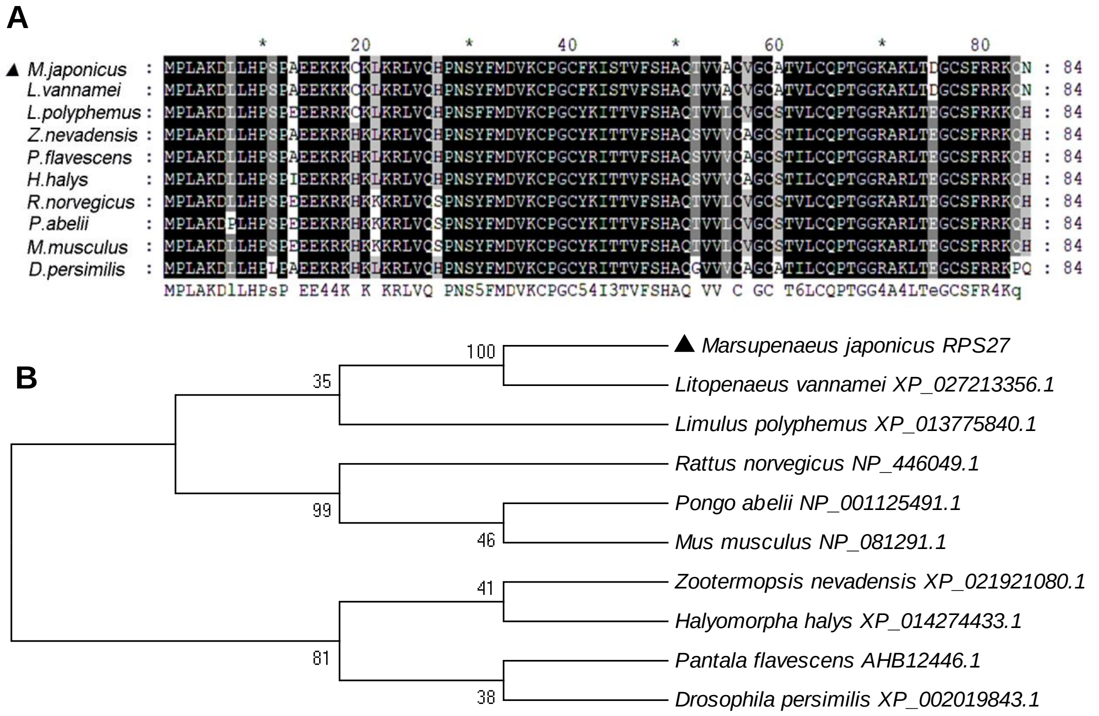

Figure S2. A, Sequence Alignment of *Mj*RPS27 with RPS27s from other species. B, Phylogenetic tree of *Mj*RPS27 and RPS27s from other species.

3. Specific mRNA silencing of *Mj*RPS27

To analyze whether the *MjRPS27* RNAi present non-specific silencing, we detected the expression of several genes by qPCR after *dsMjRPS27* injection in hemocytes and intestines of shrimp, including related gene *MjRPS28*, transcription factor genes, *Mj*Dorsal, *Mj*Relish and *Mj*FOXO, and other unrelated genes, such as *MjMthGPCR*, *MjAKT* and two small GTPases *(MjRab5* and MjRab7), and an AMP gene (*MjALF-B1*) with the primers list in Table S1.

The results showed that *MjRPS27* was successfully knocked down in hemocytes and intestine after *dsMjRPS27* injection (Figure S3 A and D). After knockdown of *MjRPS27*, we firstly analyze the expression of *MjDorsal*, *MjRelish* and *MjALF-B1*, their expression was declined significantly, suggesting that *Mj*RPS27 was related with

NF-κB pathway. To test if the *MjRPS27* knockdown is specific, we analyzed the expression of *MjRPS28*, and the results revealed that its expression was not altered comparing with the control in hemocytes and intestine of the shrimp (Figure S3C, F). Similar results were obtained for transcription factor *MjFOXO*, and other unrelated genes, *MjMthGPCR*, *MjRab5* and *MjRab7* (Figure S3C, F) in hemocytes and intestine. These results suggested that there was no non-specific silencing of the gene after injection of dsRNA of *MjRPS27*.

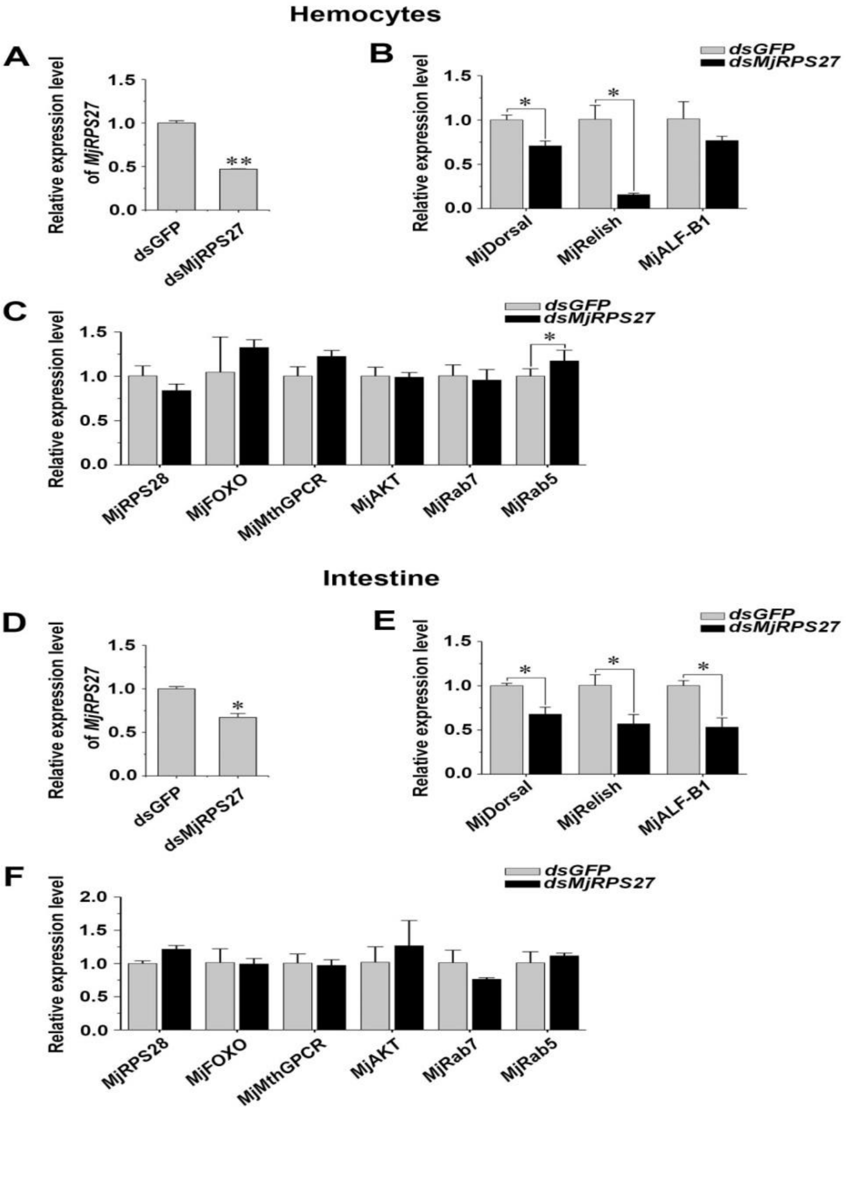

Fig. S3 Specific knockdown analysis of *Mj*RPS27 RNAi. **(A)** RNAi efficiency of *dsMjRPS27* injection in henmocytes of shrimp. **(B)** Expression analysis of *MjDorsal*, *MjRelish* and *MjALF-B1* in hemocytes of *MjRPS27*-knockdown shrimp. **(C)** Expression

analysis of *MjRPS28*, *MjFOXO*, *MjMthGPCR*, *MjRab5 and MjRab7* in hemcytes of *MjRPS27*-knockdown shrimp. Their expressions were not changed comparing with controls. **(D)** RNAi efficiency of *dsMjRPS27* injection in intestine of shrimp. **(E)** Expression analysis of *MjDorsal*, *MjRelish* and *MjALF-B1* in intestine of *MjRPS27*-knockdown shrimp. **(F)** Expression analysis of *MjRPS28*, *MjFOXO*, *MjMthGPCR*, *MjRab5 and MjRab7* in intestine of *MjRPS27*-knockdown shrimp. Their expressions were not altered comparing with controls.

4. Interaction analysis of *Mj*RPS27 and VP26

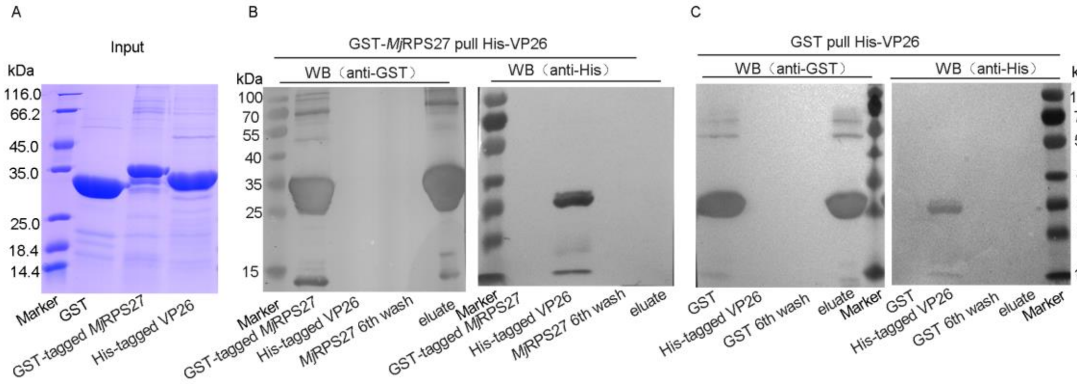

Figure S4 *Mj*RPS27 was not interacted with VP26 analyzed by GST-pulldown. (A) Input proteins of GST protein, GST-tagged *Mj*RPS27 and His-tagged VP26 analyzed by SDS-PAGE. (**B**) GST-*Mj*RPS27 pulldown His-VP26 analyzed by western blot: **Left panel**, Pulldown proteins firstly separated by SDS-PAGE and electrotransferred onto a nitrocellulose membrane and analyzed by western blot using anti-GST as primary antibody; **Right panel**, The SDS-PAGE separated proteins were transferred onto the nitrocellulose membrane and analyzed by western blot using anti-His as primary antibody. (**C**) GST pulldown His-VP26 analyzed by SDS-PAGE (control): **Left panel**, western blot analysis with anti-GST; **Right panel**, western blot analysis with anti-His.

Table S1 Primers used in the study

**Primer**	**5’– 3’ sequence**
*Mj*RPS27 RNAiF	GCGTAATACGACTCACTATAGGATTTGATGACAGACCACTTC
*Mj*RPS27 RNAiR	GCGTAATACGACTCACTATAGGATCCATTTGCCACTTTAC
GFP RNAiF	GCGTAATACGACTCACTATAGGTGGTCCCAATTCTCGTGGAAC
GFP RNAiR	GCGTAATACGACTCACTATAGGCTTGAAGTTGACCTTGATGCC
*Mj*Dorsal RTF	GCAATGCTGGTAACCTGGCTA
*Mj*Dorsal RTR	CTATGGATTTTGGTCAATACACTTT
*Mj*Relish RTF	CAGATAGATTCCTGTGCGTTGC
*Mj*Relish RTR	CGAGGTGGATTTCCGTTGTGT
*Mj*ALFB1 RTF	CGGTGGTGGCCCTGGTGGCACTCTTGG
*Mj*ALFB1 RTR	GACTGGCTGCGTGTGCTGGCTTCCCCTC
*Mj*RPS28RT-F	ATGGACAAGCCAGTAAAGC
*Mj*RPS28RT-R	ATTGCGGATAATTGAACG
*Mj*FOXO-RT-F	TTGCACCTTGATTTCCGTAG
*Mj*FOXO-RT-F	CACTTGTGGAGTCTTTCCGTAG
*Mj*Rab5-RT-F	ATTTGAGATTTGGGACACGG
*Mj*Rab5-RT-R	ATAGGTCTGGGCCTCTTCAT
*Mj*Rab7-RT-F	ATCGCGGAGCTGATTGTT
*Mj*Rab7-RT-R	ACTGTTGTGCTCGCTTCGTC
*Mj*AKT-RT-F	GTTGACTGGTGGGGTTATGGA
*Mj*AKT-RT-R	GGTGATGTAGAAGGGGTGATT
*Mj*MthGPCR-RT-F	CTCATGGGAGTCACCTGG
*Mj*MthGPCR-RT-R	CCTCGGCGTTCTCGTAG

References

1. Xu JD, Jiang HS, Wei TD, Zhang KY, Wang XW, et al. (2017) Interaction of the Small GTPase Cdc42 with Arginine Kinase Restricts White Spot Syndrome Virus in Shrimp. J Virol 91. pii: e01916-16. doi: 10.1128/JVI.01916-16.
2. Sun JJ, Lan JF, Zhao XF, Vasta GR, Wang JX (2017) Binding of a C-type lectin's coiled-coil domain to the Domeless receptor directly activates the JAK/STAT pathway in the shrimp immune response to bacterial infection. PLoS Pathog 13: e1006626. doi: 10.1371/journal.ppat.1006626.
